# Mitochondrial Respiratory Chain and Its Regulatory Elements SIRT1 and SIRT3 Play Important Role in the Initial Process of Energy Conversion after Moxibustion at Local Skin

**DOI:** 10.1155/2020/2343817

**Published:** 2020-08-21

**Authors:** Ning Zhang, Na Zhao, Lu-shuang Xie, Biao Huang, Si-rui Lin, Qun Zhang, Yuan-bing Zhu, Qiao-feng Wu, Shu-guang Yu

**Affiliations:** ^1^Acupuncture and Moxibustion School, Chengdu University of Traditional Chinese Medicine, Chengdu, Sichuan 610075, China; ^2^Institute of Acupuncture and Homeostasis Regulation, Chengdu University of Traditional Chinese Medicine, Chengdu, Sichuan 610075, China; ^3^Acupuncture & Chronobiology Key Laboratory of Sichuan Province, Chengdu, China

## Abstract

**Objectives:**

To study how thermal energy is converted after moxibustion at local skin from the view of mitochondrial respiratory chain and its key regulatory elements of sirtuins 1 (SIRT1) and sirtuins 3 (SIRT3).

**Methods:**

Two moxibustion temperatures usually used in clinical practice (38°C and 46°C) were applied to Zusanli (ST36) acupoint for 30 minutes in C57BL/6J mice. Local skin samples were harvested at 30 min and 72 h after moxibustion intervention, respectively. The activity of mitochondrial respiratory chain complexes I–V was detected by spectrophotometry. The expression of SIRT1 and SIRT3 protein was detected by immunofluorescence staining or western blot.

**Results:**

Moxibustion at 38°C triggered more significant increase of mitochondrial respiratory chain complexes I–V expression. However, the protein expression of SIRT1 and SIRT3 at 46°C showed more obvious enhancement. In addition, the effect of mitochondrial respiratory chain complexes I–V activity on local skin of ST36 acupoint was more obvious at 30 min after moxibustion, while the expression of SIRT1 and SIRT3 protein was more significant at 72 h after moxibustion.

**Conclusion:**

Mitochondrial respiratory chain and its key regulatory element proteins SIRT1 and SIRT3 play important role in the initial process of thermal energy conversion stimulated by different moxibustion temperatures in local skin.

## 1. Introduction

Moxibustion is one of the conventional thermal therapies in traditional Chinese medicine, which has been widely used in clinical practice to treat various disorders in China and other Asian countries for thousands of years [1, 2]. Although many studies have demonstrated that moxibustion has certain effects on the immune system [3], analgesia [4, 5], gastrointestinal diseases [6], etc., little is known about the initiation mechanism on conversion process of thermal stimulation induced by moxibustion. In traditional Chinese medicine theory, thermal stimulation produced during moxibustion is considered to be a vital factor in treating diseases. For example, it provides the fundamental basis for exploring the warming-dredging/promotion of *qi* and blood theory of moxibustion [7]. A study has demonstrated that the therapeutic effects of moxibustion may be possibly based on the biological effects induced by thermal stimulation [7], which is closely related to the activation of thermoreceptors and nociceptive receptors (transient receptor potential vanilloid, TRPV), heat-sensitive immune cells (Langerhans cells and their major histocompatibility complex II), and heat shock proteins (HSP) at local acupoints [8]. It could be speculated that energy conversion process of thermal stimulation might be the main influencing factor for its subsequent therapeutic effects.

The basic function of mitochondria mitochondrial respiratory chain is to promote the synthesis of adenosine triphosphate (ATP) [9, 10], which plays a crucial role in energy conversion process. Mitochondria mitochondrial respiratory chain is mainly comprised of four respiratory chain complex enzymes located on the mitochondrial intima, namely, complex I (CI), complex II (CII), complex III (CIII), and complex IV (CIV); these enzymes catalyze the oxidation of biological substrates and the synthesis of ATP. ATP synthetases are also called complex V (CV). The respiratory chain complex enzyme activity can represent the respiratory function of mitochondria directly or indirectly [11, 12]. Importantly, correlative evidence suggests that mitochondrial respiratory chain complex is also closely related to the thermal stimulation [13, 14]. SIRT1 and SIRT3 are distributed inside mitochondria [15]. Mitochondrial sirtuins are known to regulate respiratory chain function under physiological conditions and are important regulatory elements of mitochondrial energy flux [16]. Abundant evidence supports the pivotal role of SIRT1 and SIRT3 in the regulation of mitochondrial metabolism and energy homeostasis [15, 17, 18]. Thus, energy conversion related to mitochondria in local skin is one of the important ways to convert thermal energy into biological energy. Nevertheless, changes of mitochondrial respiratory chain complex enzyme activity and their key regulatory element protein SIRT1 and SIRT3 expression at local acupoints after moxibustion treatment are rarely reported.

As a kind of thermal stimulation therapy, many studies have illustrated that moxibustion with different temperatures has different therapeutic effects. During moxibustion, the temperature at local acupoints varies within a range from 34°C to 57°C [19, 20]. 38°C is close to the temperature of mild moxibustion treatment, and 46°C is close to the temperature of scar moxibustion treatment. Clinical research demonstrated that scar moxibustion temperature at 45°C or a little higher is a critical point for the therapeutic effects of moxibustion [21]. For instance, scar moxibustion could significantly reduce the blood lipids level in patients with dyslipidemia, while mild moxibustion temperature at 38°C does not have such effect [22]. Our previous animal research observed that the relationship between temperature and effect in moxibustion therapy should have a temperate-specific manner [23]. In chronic inflammatory pain, different moxibustion temperatures (from 37°C to 52°C) generated different intensities of analgesic effect: the higher the better (37°C < 42°C < 47°C < 52°C). In chronic neuropathic pain, stronger analgesic effect was found in moxibustion with temperature 47°C or 52°C rather than 37°C and 42°C [23]. Therefore, the present study aims to observe how mitochondrial respiratory chain complexes and their key regulatory element proteins SIRT1 and SIRT3 change in the initial process of moxibustion. Additionally, two usually used moxibustion temperatures (38°C and 46°C) were compared in the study, to illustrate the details of the energy conversion process induced by thermal stimulation at the local skin.

## 2. Materials and Methods

### 2.1. Animals

A total of 60 male C57BL/6J mice (6–8 weeks) weighing 18∼20 g were included in the present study. The experimental animals were purchased from Sichuan Dashuo Experimental Animal Co. Ltd. (license number: SCXK (chuan) 2017-030). Mice were housed in standard animal facilities, in which the room temperature was maintained at 24 ± 2°C and the humidity was 60–80%, with a natural 12-hour light/dark cycles (dark cycle 8 : 00 PM–8:00 AM). They had free access to water and food and were allowed to acclimate to the housing conditions for 1 week prior to the experiments. Mice were randomly divided into five groups: control group, moxi-38°C-30 min group (38°C-30 min·MB), moxi-38°C-72 h group (38°C-72 h·MB), moxi-46°C-30 min group (46°C-30 min·MB), and moxi-46°C-72 h group (46°C-72 h·MB) (*n* = 12/group).

All experimental procedures reported here were in accordance with the National Institutes of Health Guide for the Care and Use of Laboratory Animals (NIH Publications no. 80-23) revised in 1996. They also conformed to the Animal Use and Care of Medical Laboratory Animals from the Ministry of Public Health of the People's Republic of China. The study also obtained ethics committee approval from the Committee on Ethical Use of Animals of Chengdu University of Traditional Chinese Medicine (no. 2014-07).

### 2.2. Moxibustion Treatment

**Figure 1:**
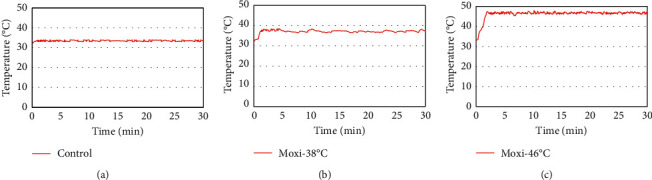
Temperature curve of local skin at ST36 acupoint in mice during moxibustion. (a) Local skin temperature curve of ST36 acupoint in control group was stable at 32 ± 1°C, while the temperature in 38°C-MB group was stable at 38 ± 1°C (b) and in 46°C-MB group was stable at 38 ± 1°C (c), respectively, after 2-3 minutes of moxibustion intervention.

Moxibustion treatment was applied to mice in 38°C-30 min·MB, 38°C-72 h·MB, 46°C-30 min·MB, and 46°C-72 h·MB group. ST36 (Zusanli) was the selected acupoint, located 2 mm lateral to the anterior tubercle of the tibia and 4 mm distal to the knee joint lower point. The location for the acupoint was determined according to Government Channel and Points Standard GB12346-90 of China and the “Veterinary Acupuncture of China.” Before the implementation of moxibustion treatment, optimal distance between animal-used moxa sticks (0.5 cm diameter, 15 cm length; Nanyang Hanyi Moxibustion Technology Development Co., Ltd., China) and skin was measured, according to the requirement of temperatures (38°C or 46°C). Moxibustion temperature at ST36 was monitored by a digital thermodetector (WZ-2300R, Xingyi Electronics Company, Hangzhou, China) and stabilized at 38 ± 1°C or 46 ± 1°C. The “paperless temperature recorder probe” was placed on the skin surface of moxibustion acupoint in all groups to monitor the real-time temperature [23]. The course of moxibustion treatment was 30 min. There was no treatment applied to mice in control group, except the test of skin temperature of local acupoint in accordance with moxibustion group.  Figure 1 shows the temperature curve of local skin during moxibustion.

### 2.3. Sample Collection

Mice in control group, 38°C-30 min·MB group, and 46°C-30 min·MB group were sacrificed after being anesthetized with 1% pentobarbital sodium (3 mL/kg), and skin tissue (approximately 1 cm × 1 cm) in ST36 was harvested. The remaining two groups (moxi-38°C-72 h group and moxi-46°C-72 h group) were sampled 72 hours later; the method was the same as before. Secondly, some of the skin tissues were immersed and fixed in a 4% paraformaldehyde solution for immunofluorescence, and others were frozen at −80°C for further western blot assay. The mitochondria in skin tissue were extracted by mitochondria extraction kit (G006, 50T, Nanjing China); the method of test was in compliance with manufacturer's protocol. The activity of mitochondrial respiratory chain complexes I–V was detected by spectrophotometry. The expression of SIRT1 and SIRT3 in skin tissue was detected by immunofluorescence or western blot.

### 2.4. Mitochondrial Respiratory Chain Complexes I–V Extraction and Activity Detection

According to the manufacturer's instructions (Mitochondrial Respiratory Chain Complexes I–V Activity Assay Kit, Solarbio Life Science, Beijing, China), every 0.1 g skin tissue was added to 1 ml extracting solution. The tissue was homogenized in the mortar on the ice. The homogenate was centrifuged at 600 g (4°C) for 10 min. The supernatant was transferred into another tube and centrifuged at 11000 g (4°C) for 15 min. Next, every 200 ∼ 600 *μ*l extracting solution was added to the precipitation and broken by the ultrasonic (power 20%, ultrasonic 5 s with 10 s interval, repeated 15 times). Then, the activity of mitochondrial respiratory chain complexes I, II, and V was determined at 340 nm, 605 nm, and 660 nm, respectively; the activity of mitochondrial respiratory chain complexes III and IV was determined at 550 nm, using a spectrophotometer in 1 ml of medium. The enzyme activity is displayed as nmol/min/mg protein [24].

### 2.5. Immunofluorescence Staining

Firstly, samples were deparaffinized in xylene, rehydrated in a series of graded alcohol, and subjected to antigen retrieval. Secondly, sections were blocked with 5% bovine serum albumin (BSA) in PBS, exposed to 0.5% Triton X-100 for 2 h, and then incubated with anti-SIRT1 (mouse monoclonal, ab110304, Abcam, UK, 1 : 200) and anti-SIRT3 (rabbit polyclonal, ab86671, Abcam, UK, 1 : 200) overnight at 4°C. The sections were washed with PBS three times and incubated with secondary antibody (Alexa Fluor 488, bs-0295G-AF488, Bioss, China, 1 : 200; excitation spectrum 495 nm, emission spectrum 519 nm, cy3, bs-0368R-Cy3, Bioss, China, 1 : 200; excitation spectrum 552, emission spectrum 570) for 2 h at 37°C. Images were captured under laser confocal microscope (FV1000, Olympus Optical Co., Ltd., Japan). The average optical density of positive expression was quantified by the Image-Pro Plus 6.0 software (Media Cybernetics, USA).

### 2.6. Western Blot

Western blot analysis was performed as previously described [25]. Whole protein extracts were prepared from mice skin tissue. A total of 20 *μ*g of protein from each sample was loaded onto sodium dodecyl sulfate polyacrylamide gel electrophoresis (SDS-PAGE) and separated by electrophoresis. The bands were transferred to a polyvinylidene difluoride membrane. Followed by blocking in 5% nonfat dry milk in Tris-buffered saline supplemented with 0.1% Tween 20 for 2 h at room temperature, the membranes were incubated overnight at 4°C with the following antibodies: SIRT1 and SIRT3 primary antibodies (used at 1 : 800 dilution). The membranes were then incubated with a secondary antibody at 37°C for 1 h. Normalization was performed by blotting the same membranes with anti-*β*-actin antibody (rabbit monoclonal, ab179467, Abcam. UK, 1 : 800). All western blot data were analyzed by Image-Pro Plus 6.0 software.

### 2.7. Statistical Analysis

All data are expressed as mean ± standard deviation ($\bar{\chi }$ ± SD). Normality was checked for all data. Differences between groups were analyzed by one-way analysis of variance (ANOVA) and post hoc test (Least-Significant Difference, LSD) with GraphPad Prism 7 (GraphPad Prism Software Inc., San Diego, USA). *P* < 0.05 was regarded as statistically significant.

## 3. Results

### 3.1. 38°C-30 min MB Group Triggered Significant Increase of Mitochondrial Respiratory Chain Complexes I–V Enzyme Expression

**Figure 2:**
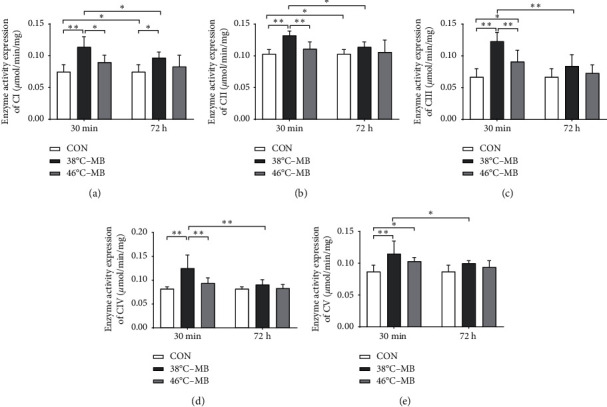
Comparison of the activity of mitochondrial respiratory chain complexes I–V enzyme after moxibustion intervention. (a–e) The expression of mitochondrial respiratory chain complexes I–V enzyme activity in ST36 local skin tissue, at 30 minutes and 72 hours after moxibustion intervention with different temperatures (^*∗*^*P* < 0.05, ^*∗∗*^*P* < 0.01, *n* = 6). MB: moxibustion; CI: complex I; CII: complex II; CIII: complex III; CIV: complex IV; CV: complex V.

Results of “30 min after moxibustion” intervention showed that the activity of mitochondrial respiratory chain complexes I–V in the local skin tissue of ST36 acupoint was elevated after moxibustion intervention. In detail, the activity of complexes I–V of mitochondrial respiratory chain in 38°C-30 min·MB group significantly increased (*P* < 0.01). In 46°C-30 min·MB group, it also increased; however, only the activity of mitochondrial respiratory chain complex enzyme III and V showed statistical significance (*P* < 0.05). Additionally, the 46°C-30 min·MB group was lower than that in the 38°C-30 min·MB group (*P* < 0.05). Results are shown in Figure 2.

Results of “72 h after moxibustion” intervention showed that, except for the activity of complex I in 38°C-72 h·MB group (*P* < 0.05), the activity of complexes I–V was slightly higher than that in the control group; however, there was no significant difference (*P* > 0.05). Furthermore, no statistical significance was found between 38°C-72 h·MB group and 46°C-72 h·MB group.

On the whole, in 38°C-MB group, the activity of mitochondrial respiratory chain complexes I–V in the local skin tissue of ST36 acupoints in “72 h after moxibustion” intervention was significantly decreased compared to that in the “30 minutes after moxibustion” intervention, but it was still slightly higher than that in the control group. In the 46°C-MB group, the activity of mitochondrial respiratory chain complexes I–V also decreased, but it was not statistically significant. Additionally, the 38°C-MB group had a continuously higher level than that in the 46°C-MB group.

### 3.2. 46°C-72 h MB Group Triggered Obvious Enhancement of the SIRT1 and SIRT3 Protein Expression

**Figure 3:**
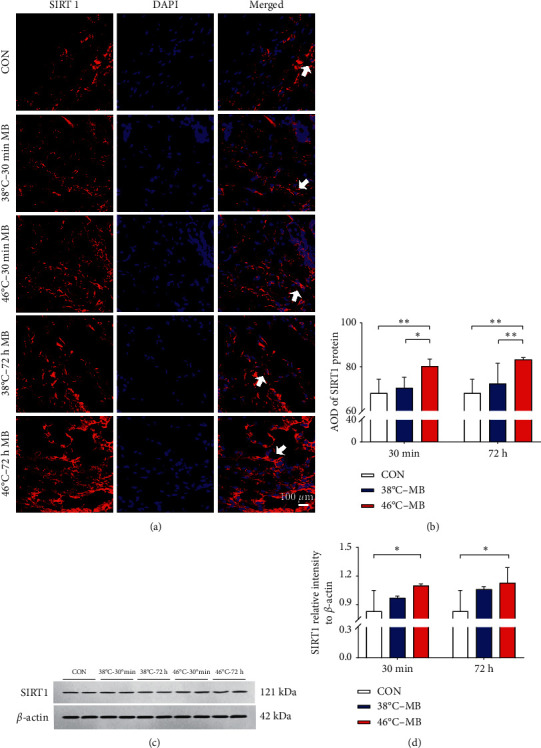
Different moxibustion temperatures have certain effect on SIRT1 protein expression in ST36 acupoint local skin tissue. (a-b) Representative labeling (a) and analysis (b) of SIRT1 protein expression in ST36 local skin tissue at 30 minutes and 72 hours after moxibustion intervention. Red SIRT1, blue DAPI nuclear staining (^*∗*^*P* < 0.05, ^*∗∗*^*P* < 0.01, *n* = 6/group). White arrow highlights the positive expression of SIRT1 protein. (c-d) Western blots (c) and analysis (d) showing the upregulation of SIRT1 protein expression in ST36 local skin tissue at 30 minutes and 72 hours after moxibustion intervention (^*∗*^*P* < 0.05, ^*∗∗*^*P* < 0.01, *n* = 3/group).

**Figure 4:**
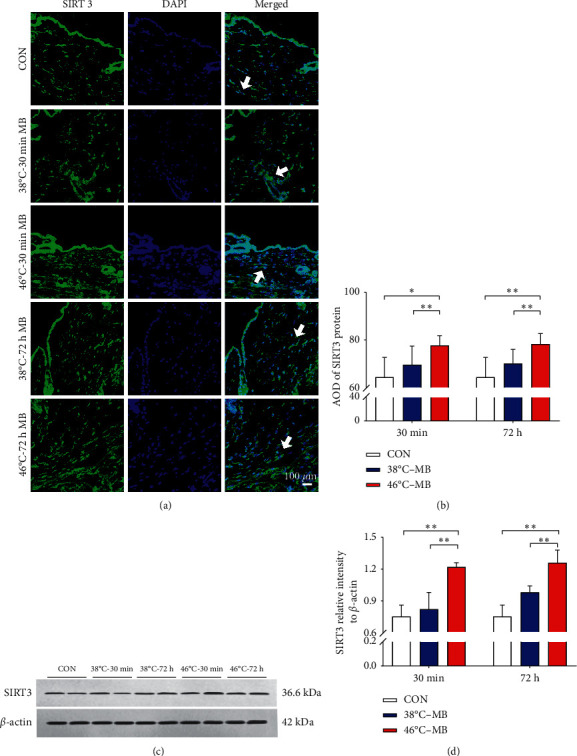
Different moxibustion temperatures have certain effect on SIRT3 protein expression in ST36 acupoint local skin tissue. (a-b) Representative labeling (a) and analysis (b) of SIRT3 protein expression in ST36 local skin tissue at 30 minutes and 72 hours after moxibustion intervention. Green SIRT3, blue DAPI nuclear staining (^*∗*^*P* < 0.05, ^*∗∗*^*P* < 0.01, *n* = 6/group). White arrow highlights the positive expression of SIRT3 protein. (c-d) Western blots (c) and analysis (d) showing the upregulation of SIRT3 protein expression in ST36 local skin tissue at 30 minutes and 72 hours after moxibustion intervention (^*∗*^*P* < 0.05, ^*∗∗*^*P* < 0.01, *n* = 3/group).

Immunofluorescence staining and analysis showed that, compared with the control group, the expression of SIRT1 and SIRT3 protein in ST36 acupoint local skin tissue indicated increasing trend after moxibustion intervention. In 46°C-30 min·MB group and 46°C-72 h·MB group, the expression of SIRT1 and SIRT3 in local skin tissue of ST36 acupoint was significantly enhanced (*P* < 0.01). In 38°C-30 min·MB group and 38°C-72 h·MB group, it also increased but exhibited no statistical significance (*P* > 0.05). Additionally, the expression of SIRT1 and SIRT3 protein in 38°C-MB group was lower than that in the 46°C-MB group with statistical significance (*P* < 0.05, *P* < 0.01). Results are shown in Figures 3 and 4.

Western blots and analysis showed that the expression of SIRT1 and SIRT3 protein in ST36 acupoint in local skin tissue also indicated increasing trend after moxibustion intervention. The expression of SIRT3 protein in local skin tissue of ST36 acupoint showed the same trend as that of immunofluorescence staining results (*P* < 0.01). However, compared with the control group, the increase of SIRT1 protein expression in 38°C-30 min·MB group and 38°C-72 h·MB group was not statistically significant (*P* > 0.05), and the expression of SIRT1 protein in 46°C-30 min·MB group and 46°C-72 h·MB group was significantly enhanced (*P* < 0.05). In particular, 46°C-72 h·MB group kept a higher level than that in 46°C-30 min·MB group. Results are shown in Figures 3 and 4.

## 4. Discussion

The effect of moxibustion, as a kind of warm stimulation, is closely related to the quantity and time of moxibustion as well as the temperature and density of moxibustion. Different moxibustion temperatures have different effects on the local microenvironment, such as blood flow, cell types and numbers, cytokines, receptors, and HSP [22, 26–28]. It is considered that suitable temperature of thermal stimulation was 40–41°C in human body since this temperature could increase the content of ATP, adenosine diphosphate (ADP), and adenosine monophosphate (AMP), enhance the cell activity, and improve the energy metabolism level of the body [26]. However, Zhu [21] reported that the moxibustion effect below 40°C was basically invalid, while the valid moxibustion temperature must reach or exceed 45°C, because it could not only activate the C-type nociceptor, but also activate the transient receptor potential vanillic acid receptor subtype I (TRPV1). Li et al. [29] compared the inhibitory effect of different moxibustion temperatures (40°C, 42°C, 44°C, 46°C, 48°C, 50°C, 52°C) on the activation of wide dynamic neurons in spinal dorsal horn induced by nociceptive colon-rectum distention. They found that 46°C∼48°C range was the best active temperature. Researchers also found that moxibustion at 46°C can significantly increase the number of mast cells (MC) in the acupoint area, followed by promoting MC degranulation, secreting chemical media, and participating in local immune response [30]. Our previous study also found that there was a temperature difference in the effect of moxibustion on the shape, distribution, and quantity of Langerhans cells at the acupoint area [28]. Therefore, revealing the details of moxibustion temperature is an important way to understand the initial mechanism of moxibustion.

In the present study, we found that different moxibustion temperatures had variable effects on the mitochondrial respiratory chain complexes I–V activity and their key regulatory element proteins SIRT1 and SIRT3 expression. This may provide a basis for energy conversion from thermal stimulation to biological effect by moxibustion intervention.

The results showed that both moxibustion at 38°C and 46°C can promote the expression of mitochondrial respiratory chain complex activity in acupoints, indicating that thermal stimulation can increase the production of mitochondrial ATP and provide energy for the body. The basic function of the mitochondrial respiratory chain is to convert the reduction potential energy generated by the substrate redox process into proton electrochemical energy, and the proton electrochemical energy can be converted into ATP [9, 10]. It is reported that 95% of the ATP in organisms comes from oxidative phosphorylation (OXPHOS) of mitochondria, and the level of mitochondrial OXPHOS affects the maintenance of energy in the body. Importantly, the mitochondrial respiratory chain plays an important role in the process of mitochondrial OXPHOS [31]. Our previous study found that there was enrichment of OXPHOS signal pathway at ST36 acupoint after moxibustion [32] and there was also a large amount of ATP release [33]. Therefore, OXPHOS may be the upstream biological process of local ATP at acupoints involved in moxibustion. Additionally, we found that moxibustion temperature of 38°C is better than 46°C on the basis of mitochondrial respiratory chain complex activity, and the immediate effect after moxibustion is better than the continuous effect (“30 minutes after moxibustion” is better than “72 hours after moxibustion”). This seems to explain our previous study results which showed that local ATP increased significantly in complete Freund᾿s adjuvant-induced chronic pain model and corresponding analgesic effect reached the peak after 45 min's moxibustion [34]. We speculated that the ATP level of the local analgesic substance in the acupoints may be related to the increase of the activity of mitochondrial respiratory chain complex enzyme after moxibustion.

Compared with 38°C-MB group, lower activity of mitochondrial respiratory chain complex and higher expression of SIRT1 and SIRT3 protein were found in 46°C-MB group. Studies have reported that higher temperatures (<52°C) cause increased membrane permeability of mitochondria, prompting exogenous H_2_O_2_ to enter the mitochondria, which results in accumulation of H_2_O_2_ in the mitochondria. However, higher mitochondrial H_2_O_2_ levels are always accompanied by lower electron transport chain complex I activity [35]. Thus, inhibition of complex I enzyme activity could lead to its electron transport barrier and then induce decreased activity of other complexes, which might be an important reason why the local mitochondrial respiratory chain complex activity expression in 46°C-MB group is lower than that in 38°C-MB group. However, further investigation is needed. SIRT3 is called “mitochondrial sirtuins” given its mitochondrial location [36]. Overexpression of SIRT3 in cells affects expression of genes involved in mitochondrial function. In light of the central role of mitochondria in many cellular processes, SIRT3 has functions aside from metabolism, for example, glycolipid metabolism, tricarboxylic acid cycle, electron transfer chain, oxidative stress, and apoptosis [37]. Furthermore, Aquilano et al. found that SIRT1 is also distributed inside mitochondria [15]. Studies have shown that the activity of SIRT1 can be enhanced with increasing temperature [38]; importantly, its activity is crucial for maintaining mitochondrial integrity and energy metabolism, alternatively, regulating cell functions including cell metabolism and cellular stress response [39, 40]. Briefly, it could be proposed that SIRT1 and SIRT3 protein may play important role in the mitochondrial integrity function after moxibustion treatment.

Studies have suggested that thermal stimulation with relatively higher temperature can induce the production of reactive oxygen species, promote the oxidation of cytochrome C, enhance the permeability of mitochondrial membrane and then promote its release into the cytoplasm, and increase the intracellular pH value, which makes the thermal control ion channel of TRPV1 open. The activation of TRPV1 can result in extracellular Ca^2+^ influx, accompanied by a small amount of Mg^2+^, K^+^, and Na^+^, which causes the release of neurotransmitters, as well as a series of biological phenomena such as cell apoptosis and mitochondrial structure and function disorders [41]. It is possible that a similar mechanism might be involved when moxibustion is at 46°C. We also found that the activity of mitochondrial respiratory chain complexes I–V in the local skin tissue of ST36 acupoint in “72 h after moxibustion” intervention was continuously at a higher level than that in the control group, and the protein expression of SIRT1 and SIRT3 was higher than that of “30 minutes after moxibustion” intervention. Therefore, the therapeutic effect of moxibustion might persist even after 72 hours.

ST36 is one of the most used acupoints in clinical practice of traditional Chinese medicine, as well as basic and clinical studies. Studies have reported that acupuncture or moxibustion at ST36 acupoint has good effects on knee osteoarthritis [42], gastrointestinal diseases [43, 44], anesthesia [45], anorexia and improving quality of life [46], etc. Electroacupuncture at ST36 acupoint can effectively inhibit lipopolysaccharide-induced multiple peritonitis in mice [47]. ST36 was also chosen to investigate the mechanism of moxibustion analgesic effect in our previous studies [23, 48, 49]. Moxibustion at ST36 acupoint can effectively increase the expression of HSP in cardiac muscle, brain tissue, gastric mucosa, and other distal tissues and organs in healthy rats [50]. According to the theory of Chinese medicine, even under healthy state, moxibustion at ST36 is often used for healthcare and enhances the body status. For instance, in ancient Chinese medical practices, it is associated with the extension of lifespan.

Hence, based on the evidence provided in current and previous studies, we could speculate that the enzymatic activities of mitochondrial respiratory chain complexes and their key regulatory element proteins SIRT1 and SIRT3 expression on local acupoint skin may participate in the initial process of energy conversion after moxibustion. These results implied that moxibustion at 38°C and 46°C will have different initial energy conversion at local skin which might induce different biological effect.

In future study, we should use antagonists to block the upregulation of “sirtuins” to verify whether the upregulation of “sirtuins” leads to upregulation of respiratory chain complex expression. More specific studies on, for example, mitochondrial marker enzymes should be detected to verify the general and specific effect on mitochondria of respiratory chain enzymes. Additionally, we did not set the intermediate time point group. It is necessary to conduct further research on the time course correlation of mitochondrial respiratory chain complexes and their regulatory element proteins SIRT1 and SIRT3 after thermal stimulation by observing the changes at several time points after moxibustion.

## 5. Conclusion

Mitochondrial respiratory chain is involved in the initial process of thermal energy conversion stimulated by different moxibustion temperatures in local skin. It could be proposed that SIRT1 and SIRT3 protein in local skin may play important role in the mitochondrial integrity function after moxibustion treatment.

## Data Availability

The data used to support the findings of this study are available from the corresponding author upon request.
